# Supplementary material for: A microRNA atlas of CD4+ T cell subsets in experimental autoimmune encephalomyelitis identifies regulators of disease pathogenesis
Source: PLoS Biol. 2026 Aug 10;24(8):e3003271. doi: 10.1371/journal.pbio.3003271 (PMC13521459; doi:10.1371/journal.pbio.3003271)
Supplement: Supplementary file 1 [file pbio.3003271.s001.pdf]

S1 Fig.

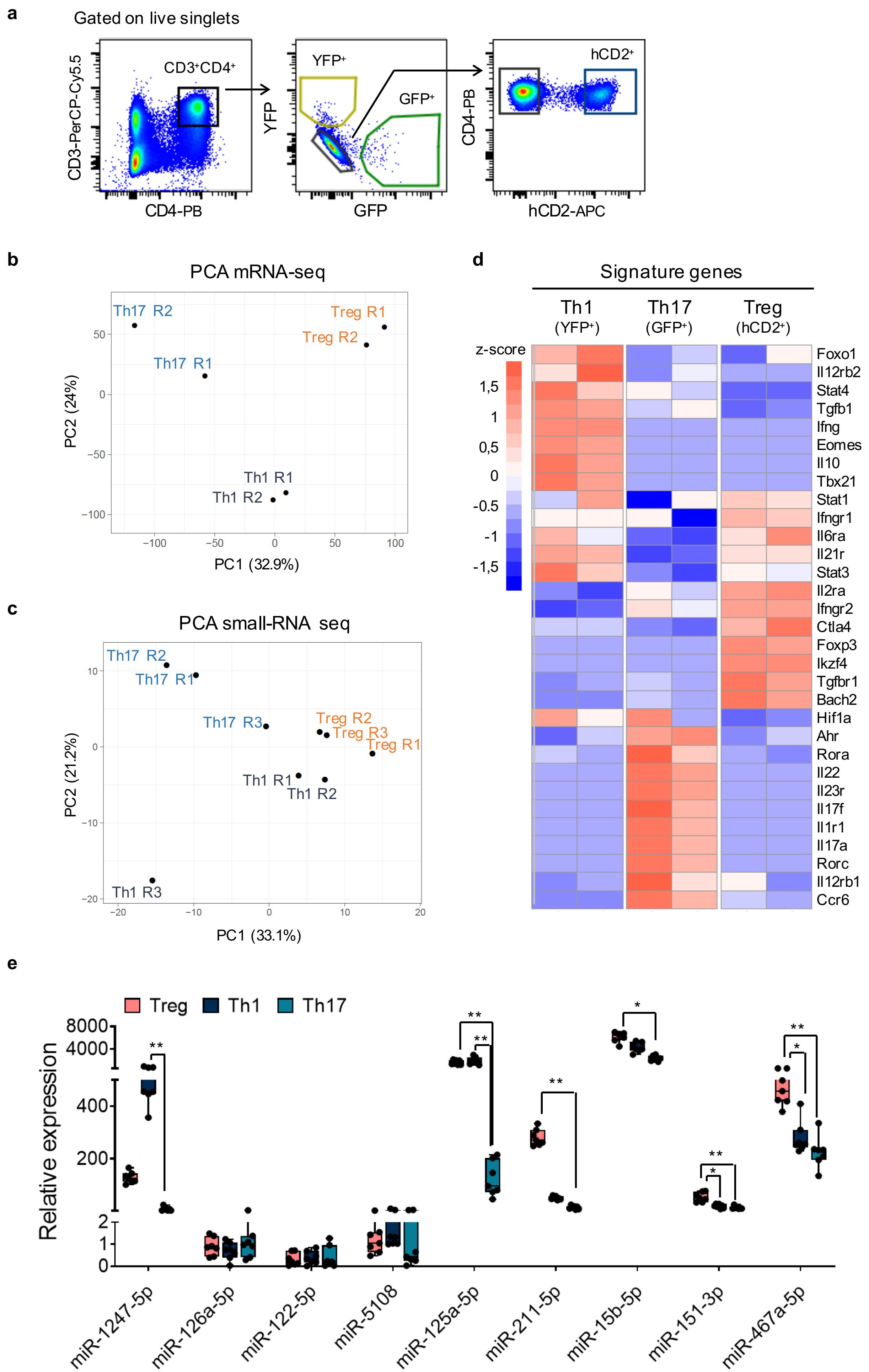
a
Gated on live singlets
CD3-PerCP-Cy5.5
CD4-PB
CD3+CD4+
YFP
GFP
YFP+
GFP+
CD4-PB
hCD2-APC
hCD2+
b
PCA mRNA-seq
Th17 R2
Th17 R1
Treg R1
Treg R2
Th1 R1
Th1 R2
PC2 (24%)
PC1 (32.9%)
50
0
−50
−100
−100
−50
0
50
100
c
PCA small-RNA seq
Th17 R2
Th17 R1
Th17 R3
Treg R2
Treg R3
Treg R1
Th1 R1
Th1 R2
Th1 R3
PC2 (21.2%)
PC1 (33.1%)
10
0
−10
−20
−20
−10
0
10
20
d
Signature genes
Th1 (YFP+)
Th17 (GFP+)
Treg (hCD2+)
z-score
1,5
1
0,5
0
-0.5
-1
-1,5
Foxo1
Il12rb2
Stat4
Tgfb1
Ifng
Eomes
Il10
Tbx21
Stat1
Ifngr1
Il6ra
Il21r
Stat3
Il2ra
Ifngr2
Ctla4
Foxp3
Ikzf4
Tgfbr1
Bach2
Hif1a
Ahr
Rora
Il22
Il23r
Il17f
Il1r1
Il17a
Rorc
Il12rb1
Ccr6
e
Treg
Th1
Th17
Relative expression
8000
4000
400
200
2
1
0
**
**
**
**
*
**
*
**
*
miR-1247-5p
miR-126a-5p
miR-122-5p
miR-5108
miR-125a-5p
miR-211-5p
miR-15b-5p
miR-151-3p
miR-467a-5p
