# Supplementary material for: A microRNA atlas of CD4+ T cell subsets in experimental autoimmune encephalomyelitis identifies regulators of disease pathogenesis
Source: PLoS Biol. 2026 Aug 10;24(8):e3003271. doi: 10.1371/journal.pbio.3003271 (PMC13521459; doi:10.1371/journal.pbio.3003271)
Supplement: Supplementary file 2 [file pbio.3003271.s002.pdf]

S2 Fig.

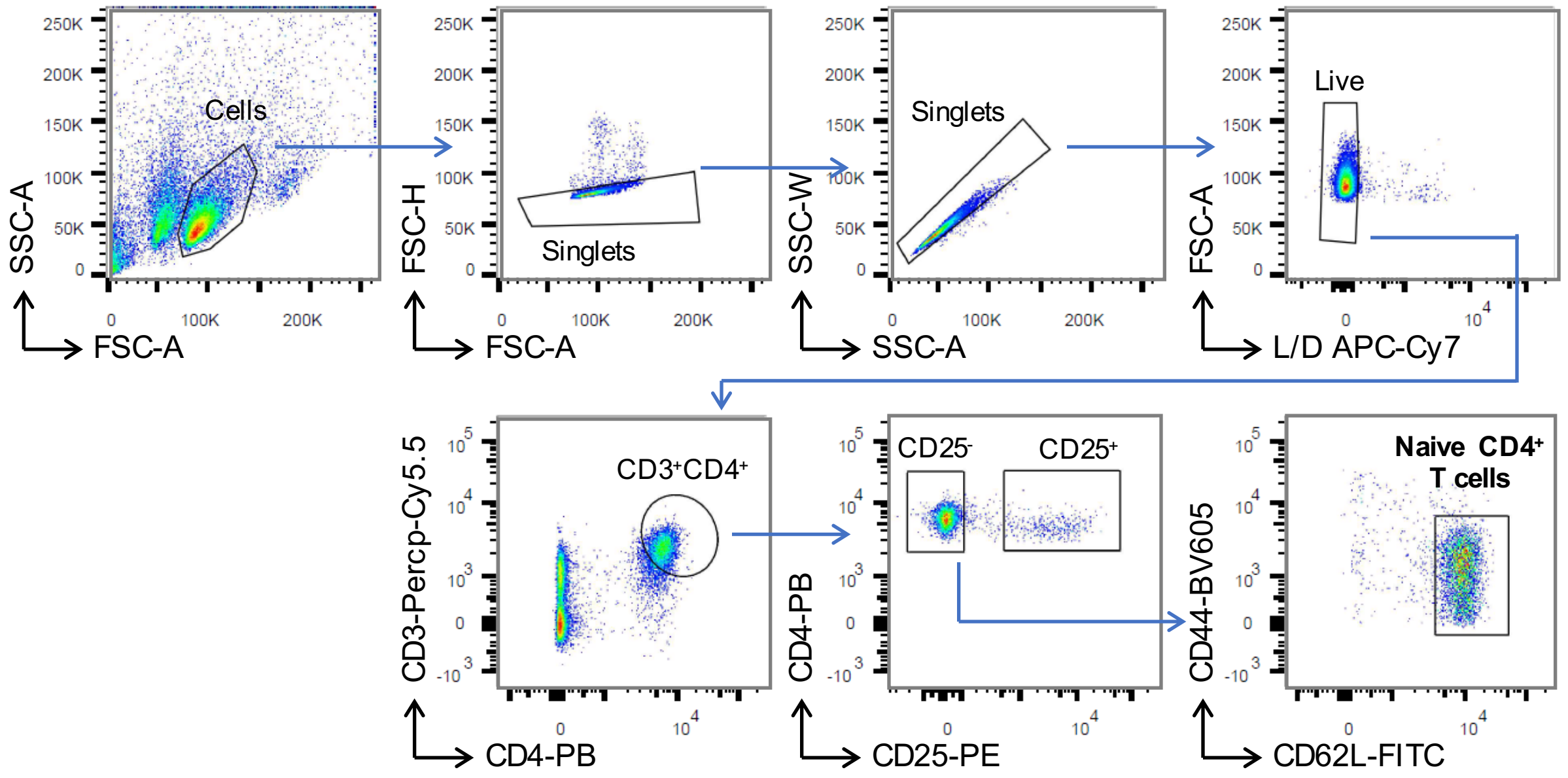

Cells
SSC-A
FSC-A
Singlets
FSC-H
FSC-A
Singlets
SSC-W
SSC-A
Live
FSC-A
L/D APC-Cy7
CD3+CD4+
CD3-Percp-Cy5.5
CD4-PB
CD25-
CD25+
CD4-PB
CD25-PE
Naive CD4+ T cells
CD44-BV605
CD62L-FITC
