# Supplementary material for: A microRNA atlas of CD4+ T cell subsets in experimental autoimmune encephalomyelitis identifies regulators of disease pathogenesis
Source: PLoS Biol. 2026 Aug 10;24(8):e3003271. doi: 10.1371/journal.pbio.3003271 (PMC13521459; doi:10.1371/journal.pbio.3003271)
Supplement: Supplementary file 3 [file pbio.3003271.s003.pdf]

S3 Fig.

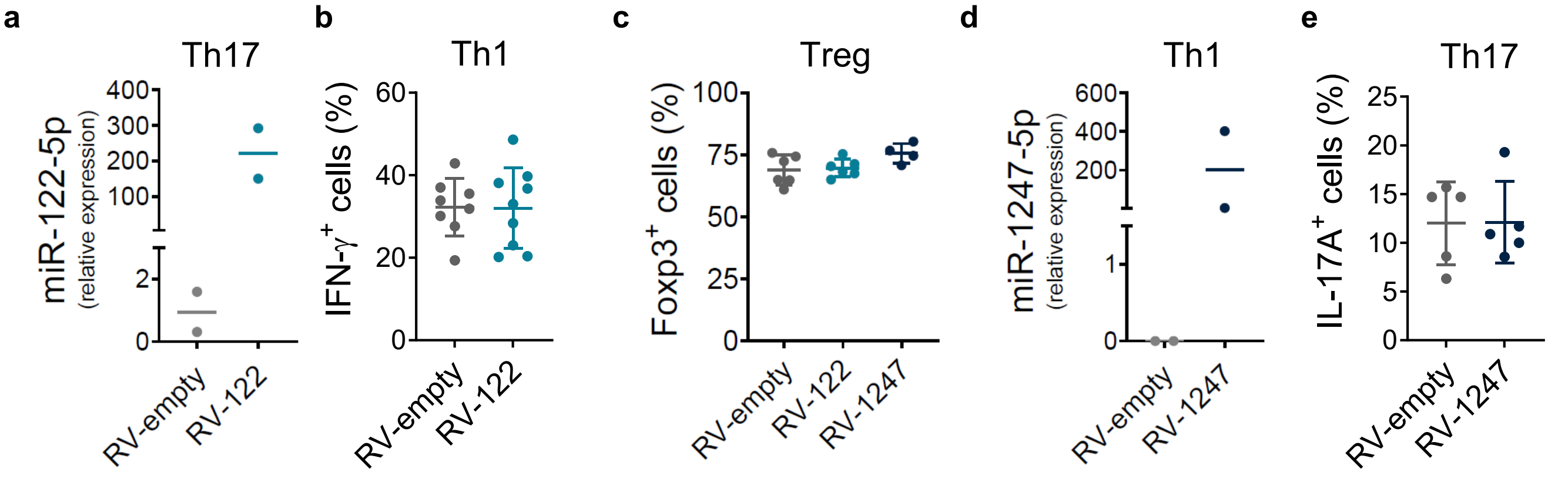
a
Th17
miR-122-5p (relative expression)
RV-empty
RV-122
b
Th1
IFN-γ+ cells (%)
RV-empty
RV-122
c
Treg
Foxp3+ cells (%)
RV-empty
RV-122
RV-1247
d
Th1
miR-1247-5p (relative expression)
RV-empty
RV-1247
e
Th17
IL-17A+ cells (%)
RV-empty
RV-1247
