# Supplementary material for: A microRNA atlas of CD4+ T cell subsets in experimental autoimmune encephalomyelitis identifies regulators of disease pathogenesis
Source: PLoS Biol. 2026 Aug 10;24(8):e3003271. doi: 10.1371/journal.pbio.3003271 (PMC13521459; doi:10.1371/journal.pbio.3003271)
Supplement: Supplementary file 4 [file pbio.3003271.s004.pdf]

S4 Fig.

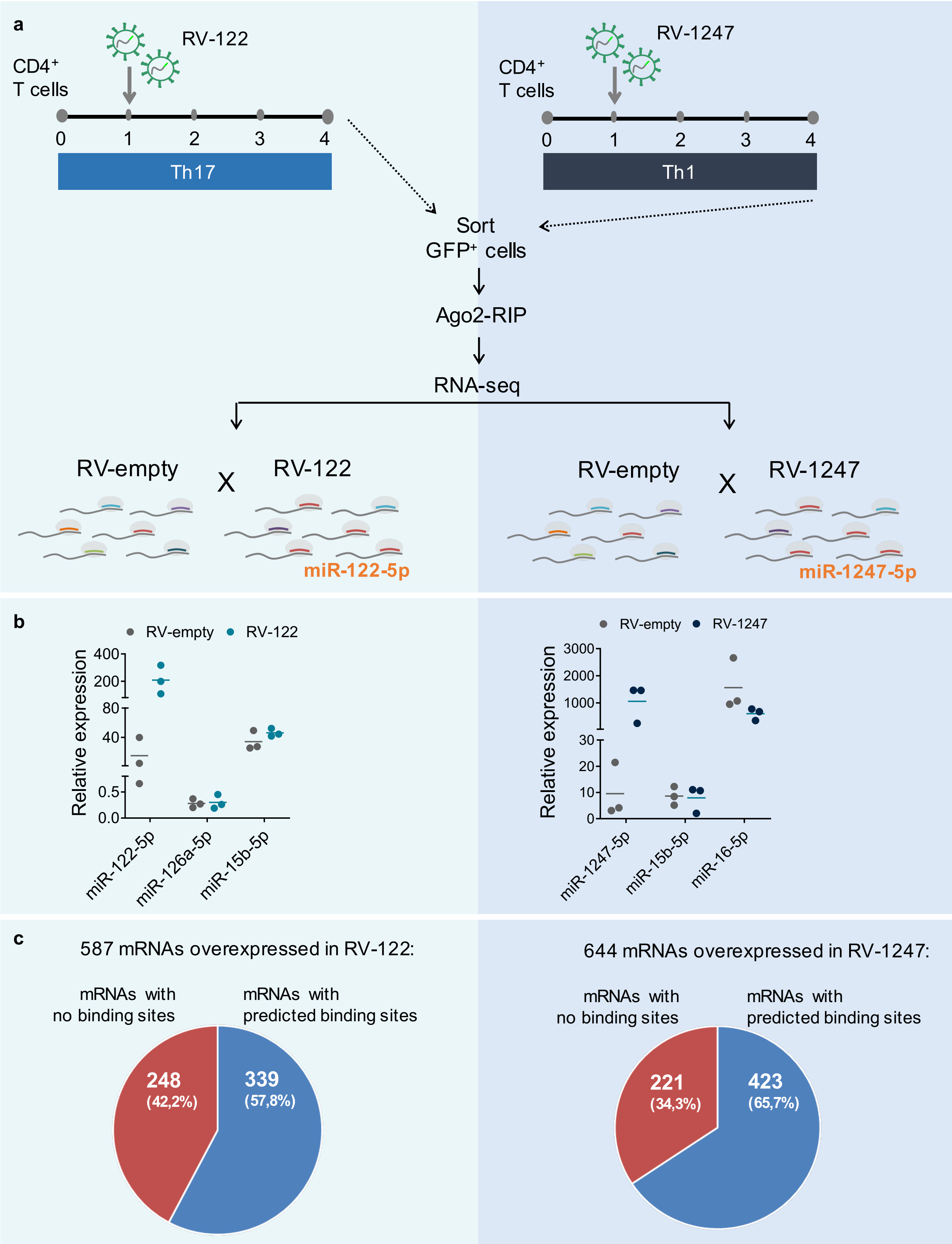
a
RV-122
CD4+
T cells
0
1
2
3
4
Th17
RV-1247
CD4+
T cells
0
1
2
3
4
Th1
Sort
GFP+ cells
Ago2-RIP
RNA-seq
RV-empty
X
RV-122
miR-122-5p
RV-empty
X
RV-1247
miR-1247-5p
b
RV-empty
RV-122
Relative expression
400
200
80
40
0.5
0.0
miR-122-5p
miR-126a-5p
miR-15b-5p
RV-empty
RV-1247
Relative expression
3000
2000
1000
30
20
10
0
miR-1247-5p
miR-15b-5p
miR-16-5p
c
587 mRNAs overexpressed in RV-122:
mRNAs with
no binding sites
mRNAs with
predicted binding sites
248
(42,2%)
339
(57,8%)
644 mRNAs overexpressed in RV-1247:
mRNAs with
no binding sites
mRNAs with
predicted binding sites
221
(34,3%)
423
(65,7%)
