# Supplementary material for: A microRNA atlas of CD4+ T cell subsets in experimental autoimmune encephalomyelitis identifies regulators of disease pathogenesis
Source: PLoS Biol. 2026 Aug 10;24(8):e3003271. doi: 10.1371/journal.pbio.3003271 (PMC13521459; doi:10.1371/journal.pbio.3003271)
Supplement: Supplementary file 6 [file pbio.3003271.s006.pdf]

S6 Fig.

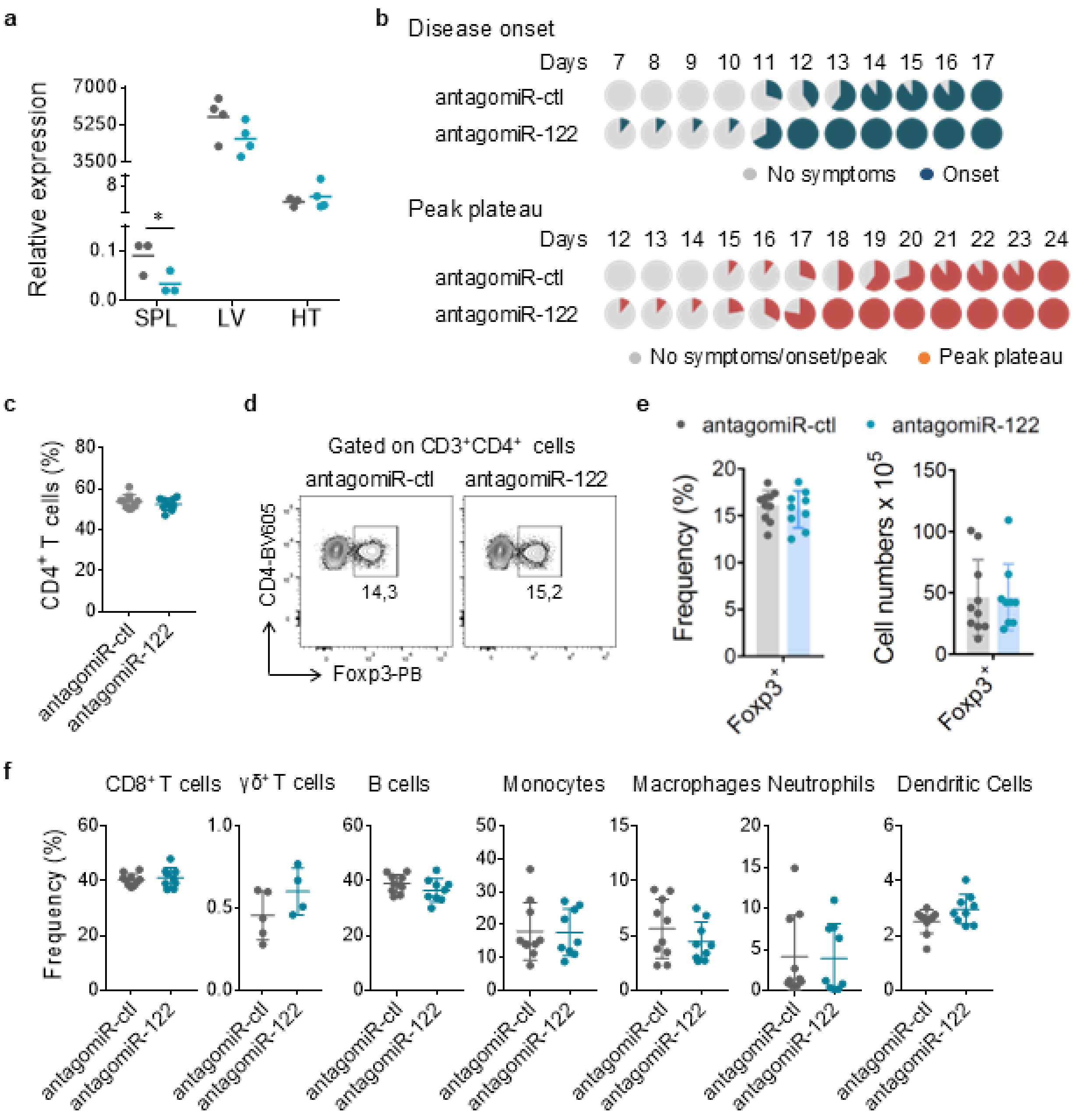

a
Relative expression
7000
5250
3500
8
0.1
0.0
*
SPL
LV
HT
b
Disease onset
Days 7 8 9 10 11 12 13 14 15 16 17
antagomiR-ctl
antagomiR-122
No symptoms
Onset
Peak plateau
Days 12 13 14 15 16 17 18 19 20 21 22 23 24
antagomiR-ctl
antagomiR-122
No symptoms/onset/peak
Peak plateau
c
CD4+ T cells (%)
80
60
40
20
0
antagomiR-ctl
antagomiR-122
d
Gated on CD3+CD4+ cells
antagomiR-ctl
antagomiR-122
CD4-BV605
Foxp3-PB
14,3
15,2
e
antagomiR-ctl
antagomiR-122
Frequency (%)
20
15
10
5
0
Foxp3+
Cell numbers x 10^5
150
100
50
0
Foxp3+
f
CD8+ T cells
γδ+ T cells
B cells
Monocytes
Macrophages
Neutrophils
Dendritic Cells
Frequency (%)
60
40
20
0
1.0
0.5
0.0
50
40
30
20
10
15
5
6
4
2
antagomiR-ctl
antagomiR-122
