# Supplementary material for: A microRNA atlas of CD4+ T cell subsets in experimental autoimmune encephalomyelitis identifies regulators of disease pathogenesis
Source: PLoS Biol. 2026 Aug 10;24(8):e3003271. doi: 10.1371/journal.pbio.3003271 (PMC13521459; doi:10.1371/journal.pbio.3003271)
Supplement: Supplementary file 7 [file pbio.3003271.s007.pdf]

S7 Fig.

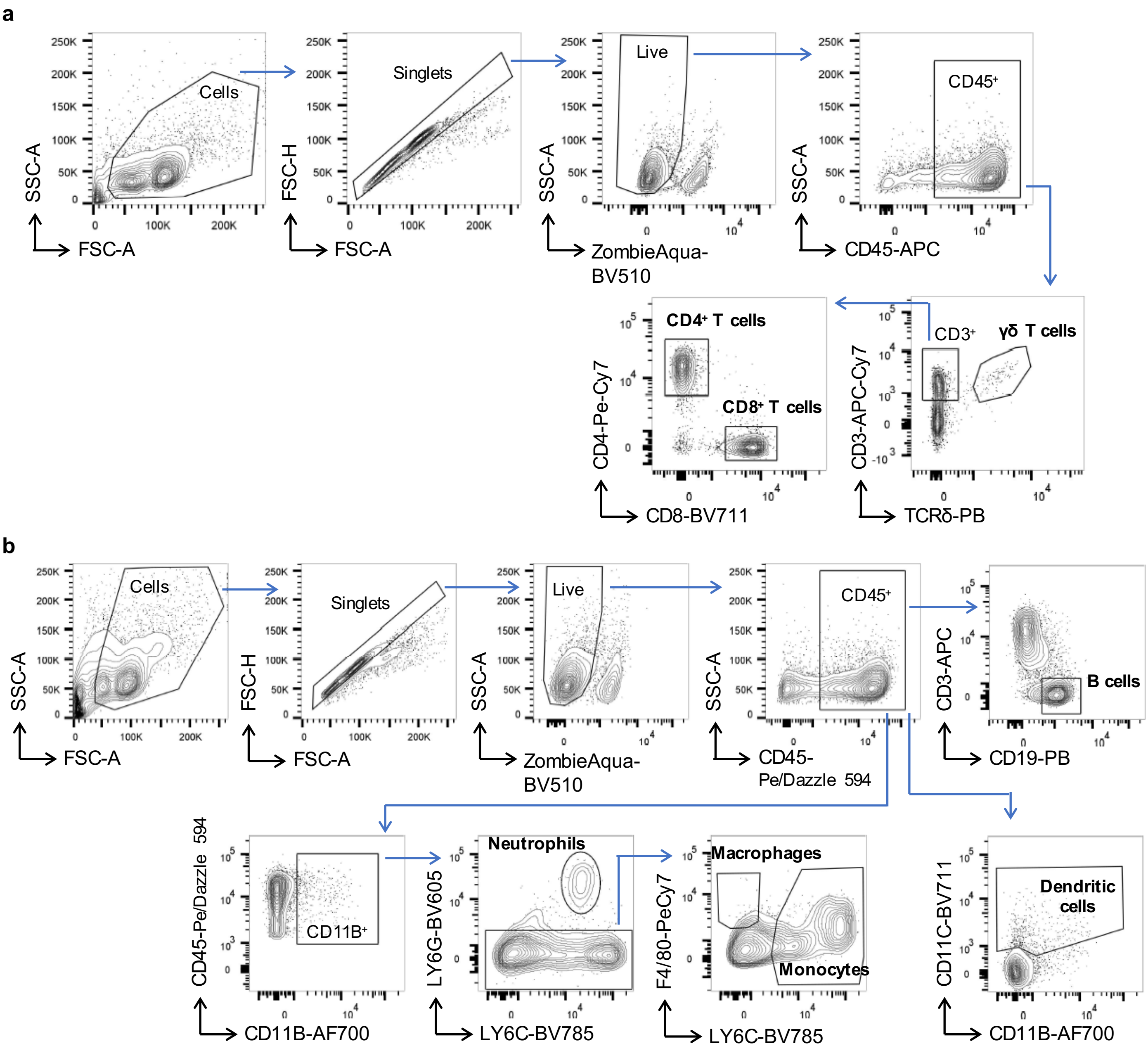
a
Cells
SSC-A
FSC-A
Singlets
FSC-H
FSC-A
Live
SSC-A
ZombieAqua-BV510
CD45+
SSC-A
CD45-APC
CD4+ T cells
CD8+ T cells
CD4-Pe-Cy7
CD8-BV711
CD3+
γδ T cells
CD3-APC-Cy7
TCRδ-PB
b
Cells
SSC-A
FSC-A
Singlets
FSC-H
FSC-A
Live
SSC-A
ZombieAqua-BV510
CD45+
SSC-A
CD45-Pe/Dazzle 594
B cells
CD3-APC
CD19-PB
CD11B+
CD45-Pe/Dazzle 594
CD11B-AF700
Neutrophils
LY6G-BV605
LY6C-BV785
Macrophages
Monocytes
F4/80-PeCy7
LY6C-BV785
Dendritic cells
CD11C-BV711
CD11B-AF700
