# Supplementary material for: A microRNA atlas of CD4+ T cell subsets in experimental autoimmune encephalomyelitis identifies regulators of disease pathogenesis
Source: PLoS Biol. 2026 Aug 10;24(8):e3003271. doi: 10.1371/journal.pbio.3003271 (PMC13521459; doi:10.1371/journal.pbio.3003271)
Supplement: Supplementary file 8 [file pbio.3003271.s008.pdf]

S8 Fig.

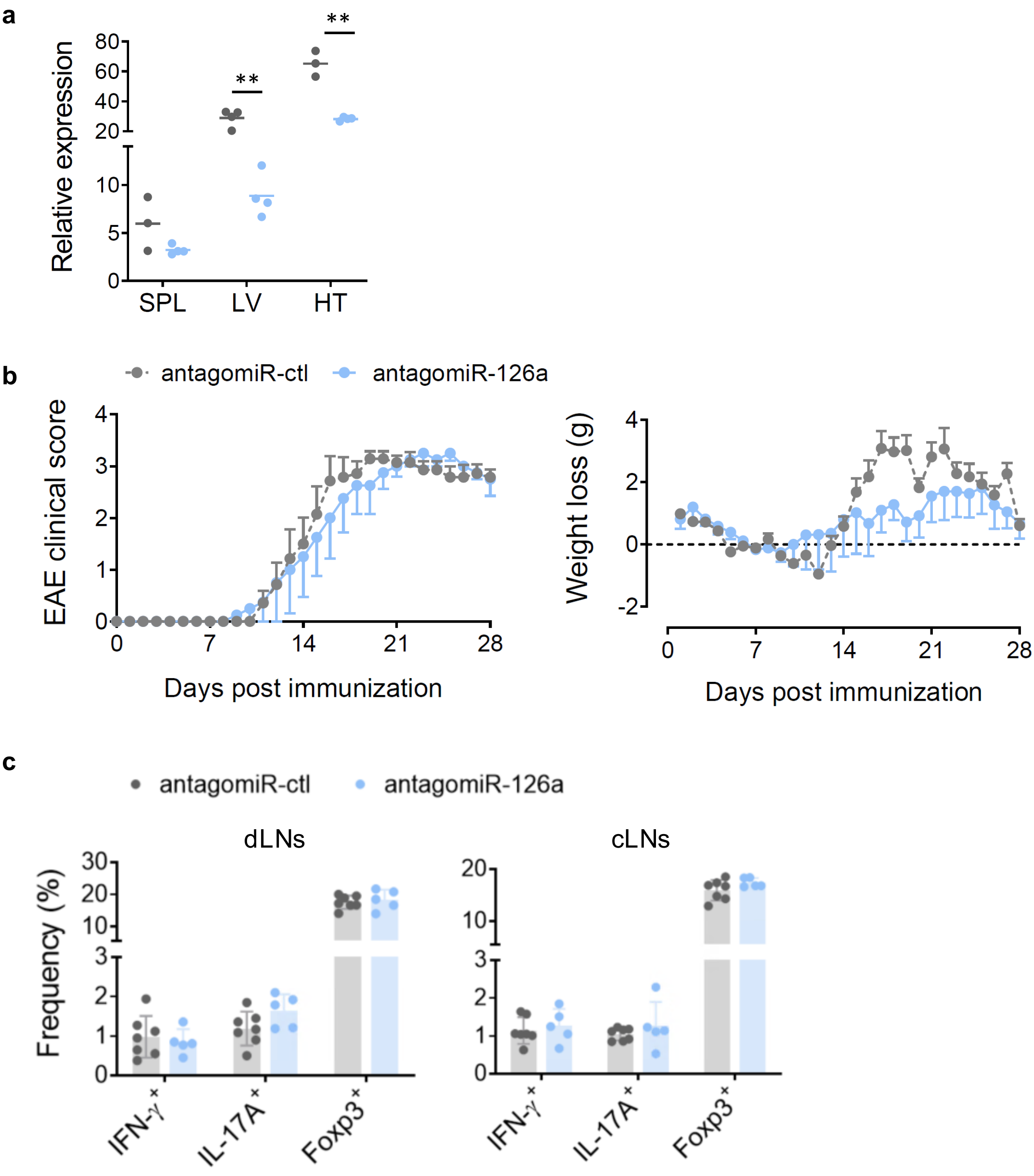
a
Relative expression
80
60
40
20
10
5
0
**
**
SPL
LV
HT
b
antagomiR-ctl
antagomiR-126a
EAE clinical score
4
3
2
1
0
0
7
14
21
28
Days post immunization
Weight loss (g)
4
2
0
-2
0
7
14
21
28
Days post immunization
c
antagomiR-ctl
antagomiR-126a
dLNs
cLNs
Frequency (%)
30
20
10
3
2
1
0
IFN-γ⁺
IL-17A⁺
Foxp3⁺
20
10
3
2
1
0
IFN-γ⁺
IL-17A⁺
Foxp3⁺
