# Supplementary material for: A microRNA atlas of CD4+ T cell subsets in experimental autoimmune encephalomyelitis identifies regulators of disease pathogenesis
Source: PLoS Biol. 2026 Aug 10;24(8):e3003271. doi: 10.1371/journal.pbio.3003271 (PMC13521459; doi:10.1371/journal.pbio.3003271)
Supplement: Supplementary file 9 [file pbio.3003271.s009.pdf]

S9 Fig.

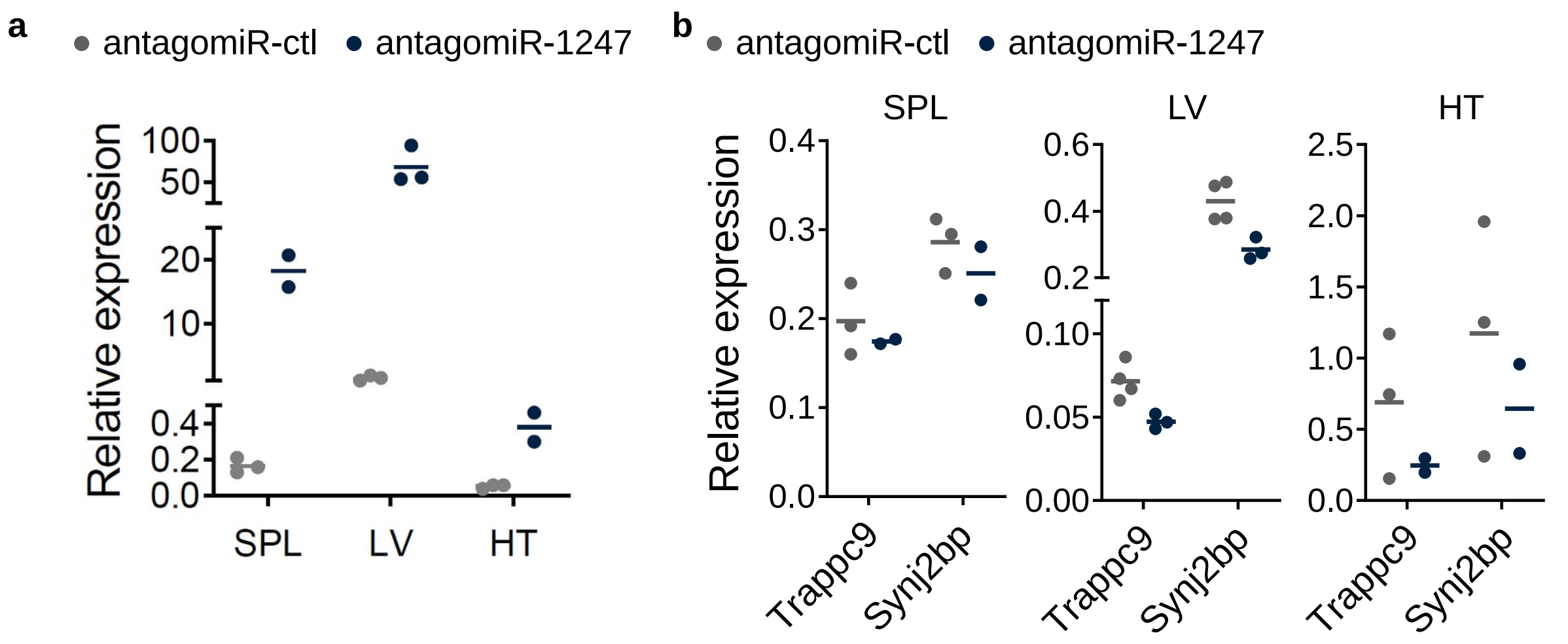
a
antagomiR-ctl
antagomiR-1247
Relative expression
100
50
20
10
0.4
0.2
0.0
SPL
LV
HT
b
antagomiR-ctl
antagomiR-1247
SPL
LV
HT
Relative expression
0.4
0.3
0.2
0.1
0.0
0.6
0.4
0.2
0.10
0.05
0.00
2.5
2.0
1.5
1.0
0.5
0.0
Trappc9
Synj2bp
Trappc9
Synj2bp
Trappc9
Synj2bp
