# Supplementary material for: A microRNA atlas of CD4+ T cell subsets in experimental autoimmune encephalomyelitis identifies regulators of disease pathogenesis
Source: PLoS Biol. 2026 Aug 10;24(8):e3003271. doi: 10.1371/journal.pbio.3003271 (PMC13521459; doi:10.1371/journal.pbio.3003271)
Supplement: Supplementary file 10 [file pbio.3003271.s010.pdf]

S10 Fig.

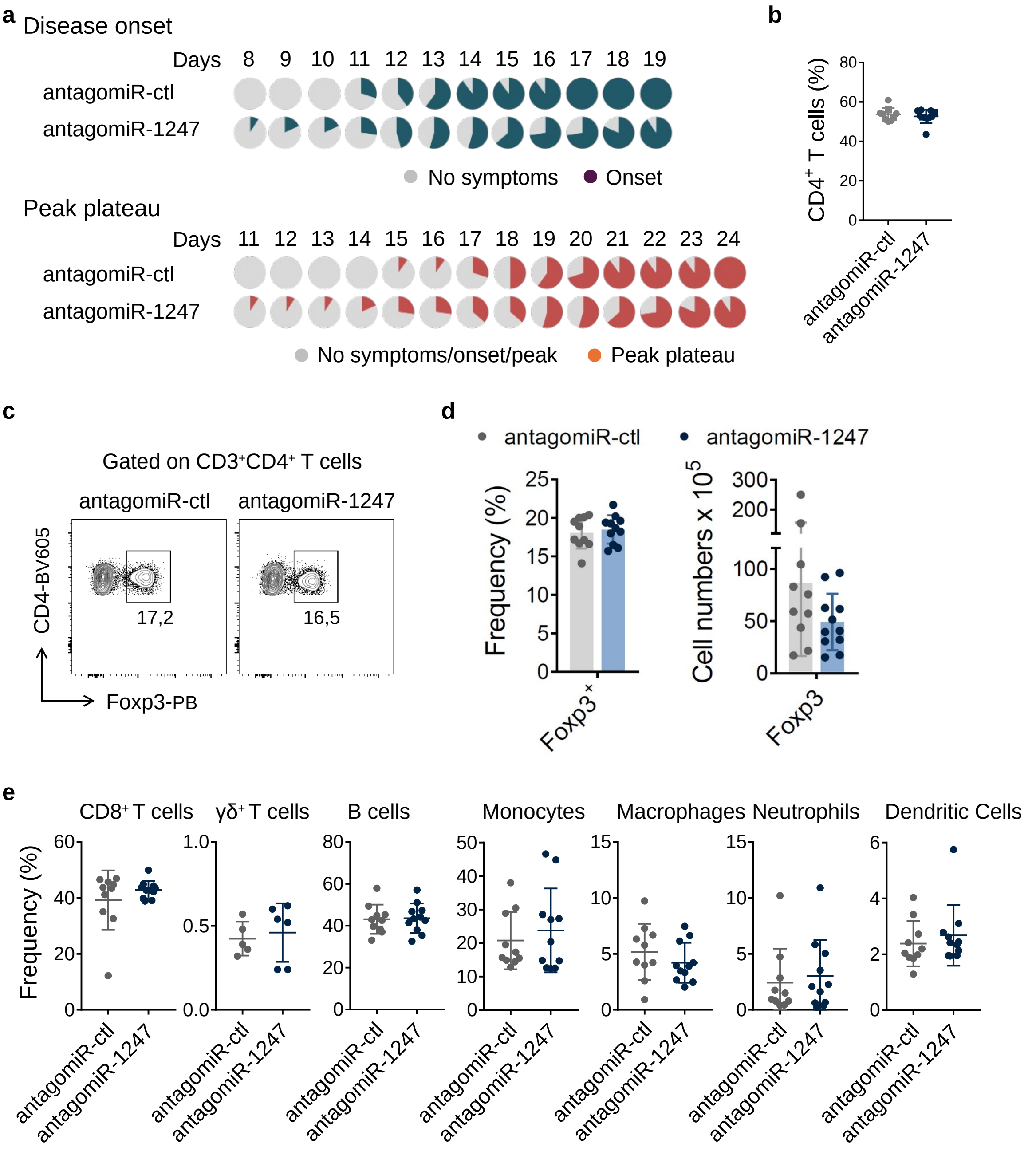
a
Disease onset
Days 8 9 10 11 12 13 14 15 16 17 18 19
antagomiR-ctl
antagomiR-1247
No symptoms
Onset
Peak plateau
Days 11 12 13 14 15 16 17 18 19 20 21 22 23 24
antagomiR-ctl
antagomiR-1247
No symptoms/onset/peak
Peak plateau
b
CD4+ T cells (%)
antagomiR-ctl
antagomiR-1247
c
Gated on CD3+CD4+ T cells
antagomiR-ctl
antagomiR-1247
CD4-BV605
Foxp3-PB
17,2
16,5
d
antagomiR-ctl
antagomiR-1247
Frequency (%)
Foxp3+
Cell numbers x 10^5
Foxp3
e
CD8+ T cells
γδ+ T cells
B cells
Monocytes
Macrophages
Neutrophils
Dendritic Cells
Frequency (%)
antagomiR-ctl
antagomiR-1247
