# Supplementary material for: A microRNA atlas of CD4+ T cell subsets in experimental autoimmune encephalomyelitis identifies regulators of disease pathogenesis
Source: PLoS Biol. 2026 Aug 10;24(8):e3003271. doi: 10.1371/journal.pbio.3003271 (PMC13521459; doi:10.1371/journal.pbio.3003271)
Supplement: Supplementary file 11 [file pbio.3003271.s011.pdf]

S1 Table

Mature.ID	Th1_SPL_R01	Th1_SPL_R02	Th1_SPL_R03	Th17_SPL_R01	Th17_SPL_R02	Th17_SPL_R03	Treg_SPL_R01	Treg_SPL_R02	Treg_SPL_R03	Th vs Treg		Th1 vs Treg		Th17 vs Treg	
	CPM									logFC	FDR	logFC	FDR	logFC	FDR
mmu-miR-211-5p	11,1987	7,3423	22,7771	14,6501	9,8020	0,0000	93,4616	217,0190	232,8192	3,7714	0,0000	3,8246	0,0001	4,0454	0,0000
mmu-miR-181b-5p	11,1987	0,0000	0,0000	0,0000	19,6039	0,0000	124,6154	19,2906	44,7729	2,5838	0,0435	2,9338	0,0514	2,0494	0,1524
mmu-miR-15b-5p	1623,8148	1534,5321	774,4218	908,3043	352,8706	353,7415	2025,0010	9027,9896	6859,2131	2,2393	0,0007	1,7896	0,0144	3,2698	0,0000
mmu-miR-151-3p	26,1304	44,0536	22,7771	29,3001	58,8118	0,0000	124,6154	154,3246	182,0766	1,8267	0,0139	1,9586	0,0247	1,9108	0,0193
mmu-miR-16-5p	10791,8356	12727,8058	1480,5124	2842,1134	2950,3900	1768,7074	13801,1604	27860,4145	25583,2531	1,7508	0,0235	1,1873	0,2325	2,9749	0,0000
mmu-miR-467a-5p	365,8249	352,4284	45,5542	161,1508	284,2568	147,3923	529,6156	1355,1630	1071,5655	1,6765	0,0060	1,5857	0,0473	2,1088	0,0004
mmu-miR-467b-5p	37,3291	36,7113	0,0000	14,6501	29,4059	0,0000	62,3077	135,0340	107,4550	1,6288	0,0413	1,4615	0,1298	2,3041	0,0152
mmu-miR-125a-5p	1791,7956	1842,9070	2596,5909	556,7026	382,2764	589,5691	2679,2320	6534,6826	6817,4250	1,5604	0,0521	0,9075	0,2521	3,2378	0,0000
mmu-miR-27a-3p	1243,0582	2129,2551	182,2169	278,3513	401,8804	29,4785	2180,7703	2488,4843	2253,5709	1,4847	0,1452	0,7775	0,4687	3,0287	0,0000
mmu-miR-16-2-3p	257,5706	370,7841	68,3313	219,7510	254,8510	0,0000	685,3849	694,4607	674,5788	1,4160	0,0435	1,2364	0,1216	1,8129	0,0108
mmu-miR-467e-5p	414,3527	253,3079	0,0000	249,0512	372,4745	176,8707	623,0772	897,0118	999,9288	1,3789	0,0586	1,5908	0,1168	1,4555	0,0088
mmu-miR-99b-5p	205,3099	312,0460	204,9940	29,3001	29,4059	88,4354	591,9234	356,8757	459,6688	1,3647	0,1336	0,6595	0,4687	3,3025	0,0000
mmu-miR-467c-5p	156,7821	99,1205	91,1085	29,3001	88,2176	58,9569	405,0002	289,3586	220,8798	1,3474	0,0707	1,0262	0,2687	2,0725	0,0056
mmu-miR-467d-5p	100,7885	106,4628	0,0000	14,6501	88,2176	58,9569	186,9232	250,7775	229,8344	1,3269	0,0716	1,1747	0,1923	1,8228	0,0152
mmu-miR-181a-5p	85,8569	73,4226	113,8856	278,3513	98,0196	294,7846	747,6927	409,9247	307,4408	1,3470	0,1336	2,2034	0,0155	0,9969	0,2376
mmu-miR-30d-5p	190,3783	231,2812	0,0000	73,2503	49,0098	147,3923	186,9232	569,0720	399,9715	1,3143	0,0985	1,0600	0,2739	2,1494	0,0029
mmu-miR-139-5p	1754,4665	1861,2626	409,9880	820,4039	607,7215	825,3968	3302,3093	3805,0661	2444,6021	1,3060	0,0368	1,0261	0,2334	1,9447	0,0000
mmu-miR-26a-5p	992,9534	1049,9430	136,6627	468,8022	362,6725	324,2630	1401,9237	1866,3632	1695,4017	1,2406	0,0632	0,9389	0,3125	1,9366	0,0000
mmu-miR-29a-3p	20889,3505	35397,0302	3598,7839	5889,3278	8292,4582	3655,3286	26543,0895	40042,4132	41632,8566	1,2270	0,1452	0,6562	0,5371	2,4367	0,0000
mmu-miR-30e-5p	115,7201	297,3615	22,7771	87,9004	49,0098	0,0000	342,6925	265,2454	289,5316	1,2238	0,1786	0,7037	0,4980	2,3054	0,0013
mmu-miR-19b-3p	5618,0258	12331,3238	250,5482	1948,4592	4499,0996	1149,6598	16013,0845	6462,3430	8235,2345	1,1825	0,2809	0,7586	0,6108	1,9081	0,0149
mmu-miR-361-5p	377,0237	477,2468	91,1085	131,8506	137,2274	0,0000	529,6156	554,6041	579,0632	1,0900	0,2106	0,5040	0,5683	-0,4301	0,6715
mmu-miR-15b-3p	1362,5112	1912,6584	455,5423	849,7040	1470,2940	913,8322	2523,4627	3240,8168	3581,8345	1,0730	0,0463	1,0694	0,1655	1,3665	0,0054
mmu-miR-3473e	455,4147	1468,4518	250,5482	556,7026	421,4843	442,1768	934,6158	2483,6617	1167,0811	0,9743	0,1654	0,7907	0,4461	1,5223	0,0097
mmu-miR-26b-5p	854,8358	759,9238	136,6627	366,2517	509,7019	117,9138	685,3849	1456,4385	1387,9609	0,9719	0,1870	0,7019	0,4548	1,6054	0,0147
mmu-miR-3473b	451,6818	1464,7807	250,5482	556,7026	411,6823	442,1768	903,4620	2459,5484	1149,1719	0,9564	0,1781	0,7706	0,4516	1,5097	0,0108
mmu-miR-27b-3p	1724,6033	2070,5170	296,1025	366,2517	617,5235	294,7846	1900,3855	2030,3331	1856,5842	0,8503	0,3221	0,2971	0,7698	1,9810	0,0001
mmu-miR-132-3p	556,2032	580,0385	273,3254	219,7510	127,4255	58,9569	218,0770	892,1891	955,1559	0,7542	0,3947	0,2034	0,8223	2,1370	0,0049
mmu-miR-21a-5p	34477,1339	34526,9725	6104,2663	18429,7871	8762,9522	4480,7254	14175,0067	51544,4192	47993,5977	0,7196	0,4352	0,2895	0,7897	1,6543	0,0390
mmu-miR-194-5p	339,6946	436,8644	68,3313	87,9004	225,4451	58,9569	311,5386	559,4267	435,7899	0,6856	0,4152	0,2958	0,7545	1,5327	0,0243
mmu-miR-342-3p	21561,2738	19831,4413	9976,3755	4321,7704	15447,8890	7988,6617	20156,5480	27291,3425	30860,4893	0,6332	0,3785	0,3149	0,6529	1,3383	0,0424
mmu-miR-30c-5p	2422,6570	3527,9554	751,6447	1054,8050	1176,2352	1090,7029	1682,3085	3650,7415	3862,4116	0,5120	0,4814	0,1744	0,8528	1,3074	0,0108
mmu-miR-128-3p	265,0364	315,7171	182,2169	58,6003	176,4353	58,9569	218,0770	438,8606	346,2440	0,4892	0,5608	0,0653	0,9398	1,5076	0,0337
mmu-miR-425-5p	500,2096	873,7288	318,8796	102,5505	235,2470	412,6984	872,3081	554,6041	671,5940	0,4806	0,6205	0,0515	0,9523	1,3966	0,0480
mmu-miR-484	1205,7291	1435,4116	1184,4099	498,1024	509,7019	648,5260	1495,3853	1687,9254	1387,9609	0,3715	0,6595	-0,0689	0,9317	1,3301	0,0046
mmu-miR-296-5p	171,7137	124,8184	68,3313	29,3001	9,8020	29,4785	62,3077	130,2114	92,5307	-0,0730	0,9485	-0,7130	0,4548	2,1110	0,0315
mmu-miR-7667-5p	41,0620	44,0536	91,1085	0,0000	0,0000	0,0000	0,0000	67,5170	29,8486	-0,0743	0,9551	-0,7454	0,5407		
mmu-miR-146b-5p	9899,6707	11835,7214	3279,9043	1728,7082	6812,3622	1238,0952	2305,3857	4403,0740	4799,6583	-0,9268	0,3199	-1,4002	0,0473	0,0615	0,9469
mmu-miR-146a-5p	85408,9241	104682,2564	30612,4400	17243,1315	52763,9507	12380,9517	19097,3167	37119,8910	42322,3597	-0,9562	0,2777	-1,4511	0,0388	0,0869	0,9226
mmu-miR-1306-5p	205,3099	198,2410	182,2169	395,5519	362,6725	235,8277	124,6154	192,9058	170,1371	-1,0961	0,1311	-0,5828	0,4891	-1,1728	0,0464
mmu-miR-150-3p	1097,4748	741,5682	774,4218	922,9544	1284,0568	795,9183	529,6156	549,7814	614,8816	-1,1374	0,0494	-0,9722	0,1961	-0,9918	0,0552
mmu-miR-130b-3p	530,0729	638,7765	865,5303	659,2531	578,3156	442,1768	373,8463	313,4719	304,4559	-1,3415	0,0378	-1,4251	0,0750	-0,7138	0,4290
mmu-miR-322-5p	145,5834	205,5832	0,0000	322,3015	362,6725	265,3061	31,1539	106,0982	152,2280	-1,4840	0,1276	-0,5648	0,6105	-1,6783	0,0068
mmu-miR-219a-5p	26,1304	36,7113	0,0000	58,6003	88,2176	58,9569	31,1539	9,6453	26,8638	-1,5152	0,1797	-0,6844	0,6105	-1,9400	0,0372
mmu-miR-744-5p	279,9681	256,9791	614,9821	322,3015	98,0196	265,3061	62,3077	135,0340	205,9555	-1,5759	0,0716	-1,8560	0,0473	-0,7549	0,3767
mmu-miR-1843b-3p	261,3035	172,5431	364,4338	1186,6556	431,2862	678,0045	155,7693	274,8907	208,9403	-1,7186	0,0650	-0,6622	0,4873	-1,9595	0,0017
mmu-miR-103-3p	246,3719	267,9925	706,0905	322,3015	205,8412	294,7846	93,4616	110,9208	202,9706	-1,7362	0,0403	-1,9724	0,0473	-1,0535	0,1297
mmu-miR-22-5p	186,6454	267,9925	113,8856	58,6003	137,2274	0,0000	62,3077	43,4038	53,7275	-1,8197	0,0435	-2,3047	0,0045	-0,7892	0,4410
mmu-miR-149-5p	164,2479	220,2678	68,3313	293,0014	107,8216	58,9569	0,0000	67,5170	74,6216	-1,8536	0,0216	-1,7664	0,0388	-1,5859	0,0637
mmu-miR-33-5p	41,0620	29,3690	22,7771	87,9004	196,0392	58,9569	31,1539	33,7585	20,8940	-1,8646	0,1038	-2,8982	0,0250	-2,3561	0,0047
mmu-miR-23a-5p	358,3591	190,8987	660,5363	131,8506	215,6431	147,3923	62,3077	159,1473	92,5307	-1,8819	0,0413	-2,3570	0,0155	-0,7649	0,3272
mmu-miR-301a-3p	253,8377	400,1531	159,4398	380,9018	901,7803	176,8707	280,3847	96,4529	32,8335	-1,9267	0,0778	-1,4284	0,2474	-2,1927	0,0371
mmu-miR-877-5p	85,8569	80,7648	182,2169	58,6003	98,0196	88,4354	0,0000	43,4038	35,8183	-1,9511	0,0235	-2,0766	0,0406	-1,4035	0,1032
mmu-let-7d-5p	7835,3729	8032,4312	20727,1729	13404,8134	10488,0972	5659,8636	2803,8475	4277,6852	4742,9459	-1,9721	0,0075	-2,1383	0,0141	-1,4783	0,0068
mmu-miR-6516-3p	350,8933	135,8318	1731,0606	820,4039	411,6823	1061,2244	124,6154	376,1662	346,2440	-1,9820	0,0707	-1,9975	0,1485	-1,4950	0,0219
mmu-miR-1247-5p	67,1923	179,8853	318,8796	14,6501	0,0000	0,0000	0,0000	24,1132	62,6821	-1,9991	0,2210	-2,7490	0,0155	2,5913	0,0943
mmu-let-7f-2-3p	52,2607	66,0803	68,3313	29,3001	88,2176	29,4785	0,0000	19,2906	20,8940	-2,0808	0,0235	-2,0717	0,0418	-1,7302	0,0852
mmu-miR-1195	26,1304	22,0268	68,3313	87,9004	58,8118	294,7846	0,0000	38,5812	29,8486	-2,0823	0,0985	-0,4239	0,7545	-2,2893	0,0124
mmu-miR-31-5p	735,3828	1123,3656	501,0965	849,7040	872,3744	530,6122	218,0770	332,7624	155,2128	-2,1342	0,0004	-2,0881	0,0033	-1,8641	0,0013
mmu-miR-2137	227,7074	249,6368	432,7651	219,7510	225,4451	854,8752	93,4616	159,1473	83,5761	-2,1696	0,0177	-1,8187	0,0414		
mmu-miR-423-5p	2881,8046	2444,9722	9270,2850	5523,0761	4508,9016	5335,6006	965,7697	1890,4765	1910,3117	-2,1887	0,0060	-2,1753	0,0269	-1,8319	0,0000
mmu-miR-143-3p	41,0620	47,7247	0,0000	29,3001	127,4255	58,9569	0,0000	4,8226	26,8638	-2,2058	0,0518	-1,5386	0,2325	-2,4224	0,0187
mmu-miR-1983	111,9872	121,1473	0,0000	43,9502	176,4353	29,4785	62,3077	24,1132	11,9394	-2,3011	0,0368	-2,2832	0,0417	-2,1957	0,0316
mmu-miR-301b-3p	14,9316	55,0669	45,5542	87,9004	58,8118	0,0000	31,1539	9,6453	8,9546	-2,3345	0,0435	-1,9943	0,1376	-2,4490	0,0189
mmu-miR-30d-3p	78,3911	33,0402	22,7771	0,0000	19,6039	0,0000	31,1539	4,8226	5,9697	-2,3505	0,0707	-2,9846	0,0155		
mmu-miR-466m-3p	3,7329	11,0134	45,5542	87,9004	9,8020	58,9569	0,0000	0,0000	14,9243	-2,4128	0,1276	-0,9250	0,6108	-2,6582	0,0316
mmu-miR-369-3p	33,5962	55,0669	0,0000	43,9502	107,8216	29,4785	0,0000	14,4679	11,9394	-2,4196	0,0235	-1,9234	0,1088	-2,6131	0,0074
mmu-miR-669c-3p	3,7329	3,6711	45,5542	29,3001	19,6039	88,4354	0,0000	14,4679	0,0000	-2,4223	0,1499	-0,1616	0,8770	-2,7598	0,0337
mmu-miR-5126	343,4275	209,2544	432,7651	366,2517	323,4647	383,2199	0,0000	120,5661	104,4702	-2,4253	0,0009	-2,3305	0,0087	-2,0871	0,0004
mmu-miR-505-5p	55,9936	11,0134	68,3313	14,6501	78,4157	29,4785	0,0000	14,4679	8,9546	-2,4552	0,0422	-2,3318	0,0910	-2,2407	0,0438
mmu-miR-3069-3p	37,3291	25,6979	250,5482	87,9004	29,4059	0,0000	0,0000	24,1132	14,9243	-2,5610	0,0575	-3,0487	0,0418	-1,4911	0,1897
mmu-miR-222-3p	489,0109	462,5623	45,5542	117,2006	264,6529	0,0000	31,1539	62,6944	50,7427	-2,5799	0,0177	-3,0415	0,0010	-1,6163	0,0835
mmu-miR-298-5p	82,1240	80,7648	273,3254	58,6003	78,4157	58,9569	0,0000	19,2906	32,8335	-2,6081	0,0103	-3,0143	0,0088	-1,6223	0,0795
mmu-miR-125b-5p	37,3291	36,7113	136,6627	73,2503	49,0098	324,2630	0,0000	14,4679	35,8183	-2,7015	0,0322	-1,7836	0,1838	-2,6738	0,0104
mmu-miR-3470b	0,0000	22,0268	45,5542	29,3001	9,8020	147,3923	0,0000	4,8226	8,9546	-2,7489	0,0901	-1,5147	0,4027	-2,8552	0,0243
mmu-miR-1895	93,3227	40,3824	364,4338	205,1010	137,2274	324,2630	0,0000	48,2264	50,7427	-2,7841	0,0133	-2,5386	0,0546	-2,4333	0,0006
mmu-miR-183-5p	343,4275	308,3749	182,2169	336,9516	303,8608	235,8277	31,1539	43,4038	71,6367	-2,8548	0,0000	-2,7557	0,0002	-2,5896	0,0000
mmu-miR-574-5p	100,7885	36,7113	227,7711	263,7012	107,8216	383,2199	31,1539	33,7585	35,8183	-2,9028	0,0103	-2,1775	0,0750	-2,9391	0,0001
mmu-miR-466q	11,1987	14,6845	68,3313	43,9502	9,8020	88,4354	0,0000	0,0000	8,9546	-2,9600	0,0381	-2,3492	0,1529	-2,9182	0,0243
mmu-miR-126a-5p	85,8569	69,7515	0,0000	600,6528	431,2862	353,7415	31,1539	53,0491	44,7729	-2,9747	0,0235	-0,7809	0,4805	-3,4955	0,0000
mmu-miR-223-3p	888,4320	1585,9279	364,4338	2124,2600	3695,3389	1827,6643	373,8463	274,8907	179,0917	-3,0369	0,0011	-2,0642	0,0141	-3,4474	0,0000
mmu-miR-33-3p	48,5278	40,3824	0,0000	117,2006	29,4059	0,0000	31,1539	4,8226	2,9849	-3,1041	0,0139	-2,8982	0,0250	-3,1922	0,0087
mmu-miR-25-5p	37,3291	22,0268	22,7771	43,9502	68,6137	29,4785	0,0000	4,8226	5,9697	-3,1437	0,0073	-2,6984	0,0418	-3,3017	0,0028
mmu-miR-130a-3p	18,6645	18,3556	91,1085	43,9502	147,0294	58,9569	0,0000	9,6453	8,9546	-3,2567	0,0133	-2,1781	0,1376	-3,4551	0,0003
mmu-miR-466i-5p	545,0045	234,9523	3689,8923	1084,1051	882,1764	2623,5826	93,4616	337,5851	229,8344	-3,3863	0,0059	-3,4130	0,0141	-2,8664	0,0000
mmu-miR-214-3p	18,6645	36,7113	136,6627	190,4509	68,6137	58,9569	62,3077	4,8226	0,0000	-3,5549	0,0235	-3,0673	0,0910	-4,0270	0,0003
mmu-miR-690	4031,5401	2881,8366	82043,1614	17697,2837	7351,4700	39206,3471	1495,3853	5121,6479	3934,0482	-3,5689	0,0137	-3,8328	0,0155	-2,7782	0,0005
mmu-miR-382-5p	37,3291	44,0536	45,5542	73,2503	68,6137	29,4785	0,0000	0,0000	8,9546	-3,5819	0,0012	-3,2668	0,0087	-3,5854	0,0007
mmu-miR-223-5p	11,1987	22,0268	0,0000	29,3001	127,4255	29,4785	0,0000	4,8226	2,9849	-3,6220	0,0153			-4,1929	0,0002
mmu-miR-145a-5p	33,5962	22,0268	0,0000	14,6501	78,4157	88,4354	0,0000	0,0000	5,9697	-3,6640	0,0063			-3,9710	0,0006
mmu-miR-221-3p	227,7074	249,6368	432,7651	395,5519	166,6333	88,4354	31,1539	19,2906	29,8486	-3,7992	0,0000	-3,9640	0,0000	-3,2579	0,0001
mmu-miR-411-5p	7,4658	44,0536	45,5542	14,6501	49,0098	0,0000	0,0000	0,0000	2,9849	-3,9055	0,0072	-3,9856	0,0141		
mmu-miR-31-3p	44,7949	73,4226	22,7771	73,2503	156,8314	58,9569	31,1539	4,8226	2,9849	-3,9220	0,0007	-3,3966	0,0051	-4,1788	0,0000
mmu-miR-182-5p	37,3291	47,7247	91,1085	14,6501	9,8020	0,0000	0,0000	4,8226	0,0000	-4,1255	0,0031	-4,7944	0,0004		
mmu-miR-147-3p	29,8633	179,8853	68,3313	14,6501	117,6235	0,0000	31,1539	0,0000	2,9849	-4,2994	0,0034	-4,7176	0,0007	-3,6638	0,0057
mmu-miR-706	294,8970	121,1473	3667,1152	981,5546	1068,4136	2564,6257	31,1539	173,6152	125,3642	-4,3189	0,0022	-4,2980	0,0086	-3,7917	0,0000
mmu-miR-1192	0,0000	0,0000	227,7711	14,6501	9,8020	147,3923	0,0000	0,0000	8,9546	-4,3191	0,0707			-3,1117	0,0260
mmu-miR-122-5p	418,0856	271,6636	728,8676	3442,7663	2058,4116	1798,1858	93,4616	77,1623	77,6064	-4,6176	0,0001	-3,0057	0,0008	-5,1012	0,0000
mmu-miR-486b-5p	1119,8723	106,4628	318,8796	483,4523	176,4353	265,3061	31,1539	24,1132	17,9092	-4,6489	0,0001	-4,9321	0,0001	-3,9607	0,0000
mmu-miR-5119	3,7329	11,0134	318,8796	263,7012	39,2078	235,8277	31,1539	4,8226	2,9849	-4,6638	0,0177	-4,3348	0,0526	-4,8326	0,0000
mmu-miR-486a-5p	1142,2697	106,4628	318,8796	483,4523	186,2372	265,3061	31,1539	24,1132	17,9092	-4,6669	0,0001	-4,9510	0,0001	-3,9788	0,0000
mmu-miR-935	59,7265	77,0937	113,8856	14,6501	9,8020	58,9569	0,0000	4,8226	0,0000	-4,8032	0,0005	-5,3969	0,0000	-3,1643	0,0491
mmu-miR-3968	7,4658	14,6845	91,1085	0,0000	9,8020	29,4785	0,0000	0,0000	0,0000	-4,8432	0,0069	-5,5891	0,0071		
mmu-miR-467f	3,7329	7,3423	45,5542	73,2503	9,8020	58,9569	0,0000	0,0000	0,0000	-5,3566	0,0046			-5,6368	0,0002
mmu-miR-451a	328,4959	40,3824	68,3313	43,9502	19,6039	58,9569	0,0000	0,0000	2,9849	-5,7537	0,0004	-6,4381	0,0000	-3,9696	0,0039
mmu-miR-144-5p	175,4467	22,0268	22,7771	43,9502	9,8020	0,0000	0,0000	0,0000	0,0000	-6,2638	0,0007	-7,2203	0,0001		
mmu-miR-598-3p	29,8633	40,3824	0,0000	0,0000	9,8020	0,0000	31,1539	4,8226	0,0000			-3,0347	0,0336		
mmu-miR-127-3p	11,1987	22,0268	22,7771	14,6501	0,0000	0,0000	0,0000	0,0000	2,9849			-3,2421	0,0473		
mmu-miR-199a-5p	0,0000	0,0000	0,0000	14,6501	9,8020	29,4785	0,0000	0,0000	0,0000					-4,2004	0,0226
mmu-miR-669h-3p	0,0000	0,0000	68,3313	14,6501	19,6039	147,3923	0,0000	0,0000	0,0000					-5,7686	0,0002
mmu-miR-5108	3,7329	0,0000	22,7771	43,9502	68,6137	88,4354	0,0000	0,0000	0,0000					-6,2302	0,0000
