# Supplementary material for: A microRNA atlas of CD4+ T cell subsets in experimental autoimmune encephalomyelitis identifies regulators of disease pathogenesis
Source: PLoS Biol. 2026 Aug 10;24(8):e3003271. doi: 10.1371/journal.pbio.3003271 (PMC13521459; doi:10.1371/journal.pbio.3003271)
Supplement: Supplementary file 12 [file pbio.3003271.s012.pdf]

S2 Table

Transcript	Log2FC	miR-binding site
**miR-122 mRNA targets**		
Th17 differentiation/ pathogenecity		
Spred2	2.46	3'UTR
Ipcef1	2.39	3'UTR (3)
Sumo3	2.34	3'UTR (4), CDS (3)
Sap130	2.12	CDS (2)
Il18rap	2.09	3'UTR
Cell activation and proliferation		
Gpr108	3.75	CDS (3)
Ap1m1	3.30	3'UTR, CDS
Gorasp2	2.51	3'UTR, CDS (2)
Pttg1ip	2.32	3'UTR (2)
Med24	2.06	CDS (3)
Mfhas1	1.94	CDS
Cdc14a	1.94	CDS (5)
Susd6	1.92	3'UTR (2)
Dtx2	1.90	CDS (8)
Akap9	1.89	CDS
Nudt4	1.61	3'UTR
**miR-1247 mRNA targets**		
Th1 differentiation and IFN-γ production		
Nlk	1.47	3'UTR
Chpf2	1.24	3'UTR (3)
Calr	1.18	3'UTR/CDS (2)
Soat2	1.12	3'UTR/CDS
Itga1	1.10	CDS
Zzef1	1.05	3'UTR (2)
Nfil3	0.88	CDS
Fnip2	0.78	3'UTR (2)/CDS
Cell activation and proliferation		
C3h2c3	2.60	3'UTR (4)
Gng2	2.50	3'UTR (6)
Tab1	2.26	CDS
Zfp324	1.49	CDS
Rasal3	1.38	3'UTR
Dctn2	1.31	3'UTR (2)
Arfip2	1.29	3'UTR (2)
Klf13	1.28	3'UTR (2)
Arhgap21	0.96	3'UTR /CDS(3)
Wipf1	0.94	3'UTR (6) /CDS (6)
