# Supplementary material for: A microRNA atlas of CD4+ T cell subsets in experimental autoimmune encephalomyelitis identifies regulators of disease pathogenesis
Source: PLoS Biol. 2026 Aug 10;24(8):e3003271. doi: 10.1371/journal.pbio.3003271 (PMC13521459; doi:10.1371/journal.pbio.3003271)
Supplement: Supplementary file 13 [file pbio.3003271.s013.pdf]

S3 Table

Gene	Exon	gRNA seq	Pam seq	CRISPOR	
				mitSpecScore	cfdSpecScore
Sap130	Exon3	TCAAGCTTCAGCCATTCCCG	TGG	85	90
Il18rap	Exon2	TGGTACATGCAACCTCGGAG	TGG	91	95
Nudt4	Exon2	CCTGGGACAATCCATTGGTC	TGG	73	91
Rasal3	Exon4	CCGAACATTGTGGACCTGGT	TGG	88	95
Gng2	Exon2	TGACTTGATGGCCTACTGTG	AGG	72	79
Cd5	Exon3	TCTTCTGCCAAGGATCTCCG	*from Guruprasad et al., Nature Protocols (2024)*		
