# Supplementary material for: A microRNA atlas of CD4+ T cell subsets in experimental autoimmune encephalomyelitis identifies regulators of disease pathogenesis
Source: PLoS Biol. 2026 Aug 10;24(8):e3003271. doi: 10.1371/journal.pbio.3003271 (PMC13521459; doi:10.1371/journal.pbio.3003271)
Supplement: Supplementary file 14 [file pbio.3003271.s014.pdf]

S4 Table

Gene	Sequence	
	Fwd primer	Rev primer
Zfp324	TCTGCCGCAACTCACATCTT	AAAGGCTTCTCTCCGGTGTG
Itga1	ATGACGCTCTGCCAAACTCA	TGTTGTACGCACTGTCTCCC
Gng2	GGAAGCCAACATCGACAGGA	AGGGTCTTCCTTGGCATGTG
Arfip2	ACGGACGGGATTCTAGGGAA	GGTCCTGACACCATCACCTG
Zzef1	CAACCCAGCAGGAGTCTGAG	CTCTGCTGTAGCTCGCTCTC
Chpf2	CGGAGTCTCCTATTGCGCTT	AGGGCTCTCCTCCTTCTCAG
Tab1	TCTGGAGTTGGTTCAGCGTC	GCCATTGAAGACCCCGTACA
Fnip2	AAAGAAGCGAGCAGAGCAGT	TAGAGCTGGCAGAGGCACTA
Klf13	CGCACCTGAGAACTCACACA	GATGGGGCAGCTGAACTTCT
C3h2c3	GCAAAGGAACAGCTGGCATC	TCGAGTCACATTCCCAGTGC
Soat2	CTGCGGTTTGGAGACAGGAT	GACACCAGGAACACTCCCAG
Dctn2	GGTACAGCGACTTGTCACCA	GTGGCCAAGTTTTCACGCAT
Calr	CCTGAATACTCCCCCGATGC	GCTTCTCTGCAGCCTTGGTA
Nfil3	ACCGCACAAGCTTCGGATTA	GTTCGTCACCTGCACTGAGA
Rasal3	GACCTAGGTGCTGAGAGGGA	GAGAAACAGAGGCTCCCACC
Arhgap21	CCCCAGAGTCTGCAATCCAG	GGCCCTCCTTCCTTCACTTG
Wipf1	CCCACCCTCCACATCAGTTC	CTGTTGGATCCACTTCGGCT
Nlk	ACAGCCATCTCTTCCTGTGC	CTCAGCCGCCCTTCATCTAG
Cdc14a	CCAGTACACCAGAAGCAGCA	AGGAGGAAGAGAGTGACCCC
Pttglip	GGTGAACTTCGAGGCCTTGA	CACTCTCCTCTCCTCCTGCT
Nudt4	TGAGTAGCAGTCGGTACCCA	CGAGCAGTCTGCCTAGCTTT
Gorasp2	ACCTACACGCCCCTTTGAAG	GCCGAGCTAATGGAGAGTCC
Susd6	GGGGGTTTCTGGGGATCAAG	CTTGGCATGTTCCTCCCACT
Ap1m1	TCCTCTTCGACAACACAGGC	TCGGGTGGGATGAAGGAGAT
Akap9	TGCTCTCTCTCTCCAGTCCC	AAGTTCACCTCGCCAGTCTG
Il18rap	TATTGGCCCCACAGACGAAC	GCAGCTGAGACTGGGACAAT
Spred2	TCGTGTGACACAAGCGATGA	ACCTGCACATCACTCCACAG
Ipcef1	CTGAGCAGGTCCACACACTT	GATCCAGCAGCTCTGTCTGG
Sap130	GAGAAGCAGGAGCCAGTTGT	CTAGGCATGGTAGGCTTCGG
Gpr108	TCGCCATTCTGCTGCAAGTA	TCCTCCTCATCCTCCTGTGG
Dtx2	GCCACTGTCTGCTCCTTCAT	CCTCATGGTGCCAGTGTTCT
Med24	AGATGGTGTCCTGCTCCTCT	GCAACTGAGTCGGTCACAGA
Mfhas1	GCCAGTTTGGAGAGCCTCAT	GGCACTGAGGTGAGCTGATT
Sumo3	GGCTCGGTGGTACAGTTCAA	GGGCTGGAGTGTCTGTTTCA
Actb	CGTGAAAAGATGACCCAGATCA	TGGTACGACCAGAGGCATACAG
Hprt	GACCTCTCGAAGTGTTGGAT	GCTTTGTATTTGGCTTTTCC
B2m	CTGCAGAGTTAAGCATGCCAGTAT	ATCACATGTCTCGATCCCAGTAGA
